# 

**Published:** 1863-02

**Authors:** 


### CONTENTS.

ORIGINAL ESSAYS AND COMMUNICATIONS.

Proximal Cavities. By Dr. J. S. Latimer .......... 55
Does Breathing Through the Mouth Injure the Teeth? By Geo. F. Foote, M. D.......... 60
Success in the Dental Profession. By C. C. Dills, D. D. S.......... 63
Metals and Alloys. By Dr. B. Wood.......... 70
Retrospective and Apologetic. By C. C. Dills, D. D. S.......... 75

PROCEEDINGS OF SOCIETIES.

The Cincinnati Dental Society.......... 77
Kalamazoo, Michigan, Dental Society.......... 80

CORRESPONDENCE.

"Frankness—Honesty—Truth" A Review. By J. D. Anderson, D. D. S.......... 83

SELECTIONS.

Dentition and its Derangements. By A. Jacobi, M. D.......... 86
Treatment of Diseases by Oxygen Gas.......... 91
District Court—Judge Hare.—The Use of Chloroform.......... 95

EDITORIAL.

Cincinnati Dental Association.......... 98
Fee Bill.......... 98
Obituary.......... 99

